# Spontaneous Resolution of Uncomplicated Appendicitis may Explain Increase in Proportion of Complicated Appendicitis During Covid-19 Pandemic: a Systematic Review and Meta-analysis

**DOI:** 10.1007/s00268-023-07027-z

**Published:** 2023-05-04

**Authors:** Roland E. Andersson, Maria Agiorgiti, Marcus Bendtsen

**Affiliations:** 1 Department of Surgery, County Hospital Ryhov, Box 1024, SE 551 11 Jönköping, Region Jönköpings Län Sweden; 2 Futurum, Academy for Health and Care, Jönköping, Region Jönköpings Län Sweden; 3 Department of Biomedical and Clinical Sciences, Linköping University, Linköping, Sweden; 4 Bra Liv Eksjö Primary Care Centre, Eksjö, Region Jönköping County Sweden; 5 Department of Experimental Physiology, Faculty of Medicine, University of Ioannina, Ioannina, Greece; 6 Department of Health, Medicine and Caring Sciences, Linköping University, Linköping, Sweden

## Abstract

**Background:**

Reports of an increased proportion of complicated appendicitis during the Covid-19 pandemic suggest a worse outcome due to delay secondary to the restrained access to health care, but may be explained by a concomitant decrease in uncomplicated appendicitis. We analyze the impact of the pandemic on the incidences of complicated and uncomplicated appendicitis.

**Method:**

We did a systematic literature search in the PubMed, Embase and Web Of Science databases on December 21, 2022 with the search terms (appendicitis OR appendectomy) AND (“COVID” OR SARS-Cov2 OR “coronavirus”). Studies reporting the number of complicated and uncomplicated appendicitis during identical calendar periods in 2020 and the pre-pandemic year(s) were included. Reports with indications suggesting a change in how the patients were diagnosed and managed between the two periods were excluded. No protocol was prepared in advance. We did random effects meta-analysis of the change in proportion of complicated appendicitis, expressed as the risk ratio (RR), and of the change in number of patients with complicated and uncomplicated appendicitis during the pandemic compared with pre-pandemic periods, expressed as the incidence ratio (IR). We did separate analyses for studies based on single- and multi-center and regional data, age-categories and prehospital delay.

**Results:**

The meta-analysis of 100,059 patients in 63 reports from 25 countries shows an increase in the proportion of complicated appendicitis during the pandemic period (RR 1.39, 95% confidence interval (95% CI 1.25, 1.53). This was mainly explained by a decreased incidence of uncomplicated appendicitis (incidence ratio (IR) 0.66, 95% CI 0.59, 0.73). No increase in complicated appendicitis was seen in multi-center and regional reports combined (IR 0.98, 95% CI 0.90, 1.07).

**Conclusion:**

The increased proportion of complicated appendicitis during Covid-19 is explained by a decrease in the incidence of uncomplicated appendicitis, whereas the incidence of complicated appendicitis remained stable. This result is more evident in the multi-center and regional based reports. This suggests an increase in spontaneously resolving appendicitis due to the restrained access to health care. This has important principal implications for the management of patients with suspected appendicitis.

**Supplementary Information:**

The online version contains supplementary material available at 10.1007/s00268-023-07027-z.

## Introduction

A large number of studies and meta-analyses have reported an increase in the proportion of complicated appendicitis during the Covid-19 pandemic, suggesting increased delay as explanation [1–7]. Some studies also report a concomitant decrease in the number of uncomplicated appendicitis, suggesting that undiagnosed uncomplicated appendicitis may resolve without treatment due to the restrained access to health care [4]. As the proportion of complicated appendicitis is determined by the number of complicated appendicitis as numerator and the total number of patients with appendicitis as denominator, the concomitant decrease in the number of patients with uncomplicated appendicitis must also have had an impact [4]. It is known that variations in the incidence rate of uncomplicated appendicitis are more closely associated with the proportion of complicated appendicitis [8], whereas the impact of the Covid pandemic on the proportion of complicated appendicitis has been analyzed in several meta-analyses we have not seen any previous analysis of the impact on the incidences of complicated and uncomplicated appendicitis separately [2–7].

The aim of this report is to analyze the impact of the Covid-19 pandemic on the incidence of complicated and uncomplicated appendicitis. The hypothesis is that an increase in the proportion of complicated appendicitis during the pandemic period may be the secondary effect of a decrease in the incidence of uncomplicated appendicitis rather than an increase in the incidence of complicated appendicitis [9]. This would suggest that cases of uncomplicated appendicitis were allowed to resolve undetected.

## Methods

### Study selection and search strategy

We follow the PRISMA (preferred reporting items for systematic reviews and meta-analysis) guidelines [10]. The PubMed, Embase and Web Of Science databases were searched on December 21, 2022 with the search terms (appendicitis OR appendectomy) AND (“COVID” OR SARS-Cov2 OR “coronavirus”). We also included all references from previously published meta-analysis on this issue to the search.

After removal of duplicates from the search strategy, titles and abstracts were screened for potentially eligible reports, which were reviewed in full text. RA and MA independently reviewed the records and reports. RA is responsible for the final selection. We included studies reporting number of patients with uncomplicated and complicated appendicitis treated within the 1st wave of the COVID-19 pandemic and the corresponding numbers during a reference period with the corresponding calendar dates in the preceding year(s), to control for seasonality. For studies reporting number of patients from more than one pre-pandemic year we used the mean numbers for these years. If result was reported as percentage the exact numbers were estimated from the percentages and total numbers. Studies with available full text in English, German, or Scandinavian language were eligible for selection.

Reasons for not including a report were: unclear definition of complicated and uncomplicated appendicitis [11, 12], indications suggesting change in referral area during the pandemic [13, 14], and inconsistent or incomplete data [15, 16]. Studies reporting similar number of patients treated non-operatively with antibiotics in the two periods were included but studies that only reported results from non-operative management or with strong increase in use of non-operative management for assumed uncomplicated appendicitis during the pandemic period were excluded [17–20]. Two studies with extremely small samples were also excluded [21, 22]. After exclusions there remained 63 reports for the meta-analysis. The study selection process is pictured in the PRISMA flowchart (Supplementary Fig. 1).

### Data items

The included studies report on retrospective cohorts of patients treated for appendicitis during the pandemic and a reference period identified through administrative registers. We classified the outcome as complicated and uncomplicated based on the description of the severity in the reports. However, the classification of the grade of severity of appendicitis is not uniform between the reports. Some use the ICD codes only but most refer in general terms to information from imaging (to detect abscesses or phlegmon), perioperative findings or histopathologic examination of the specimen. Only a few studies mention histopathologic depth of inflammation or presence of necrosis in general terms but with no details on the criteria used for the grading of severity. Many studies just use terms like “simple,” “uncomplicated,” “complicated,” “perforated,” “abscess” or “phlegmon” without reporting the criteria used for the classification. Patients that had been treated non-operatively for abscess or phlegmon are classified as complicated and patients treated non-operatively for assumed uncomplicated appendicitis as uncomplicated. The classification of gangrenous appendicitis as complicated or uncomplicated varies between the studies. We have assigned gangrenous appendicitis as complicated if the numbers are reported. Some studies use only the ICD-10 codes for the classification of severity, which has been criticized for unclear definitions [23]. Some classify K35.3 (Acute appendicitis with localized peritonitis) as complicated and others as uncomplicated.

We have classified the reports related to the age categories as children, adults, and all ages. The definition of children varied from 12 to 18 years. If age was not specified, we classified the report as representing all ages. The study base for the reports is classified as single- or multi-center, and as regional for reports based on larger population based registers. We extracted data on delay before arrival to hospital from information on the time from onset of symptoms to hospitalization or operation. If this duration of symptoms differed significantly between the pandemic and the pre-pandemic periods we accepted this as an indication of delay associated with the Covid-19 pandemic, classified as “1” if significant and “0” if not significant. Too few reports give information on the use of diagnostic imaging for a meaningful analysis. Due to the heterogeneity of the reports and incomplete data on various factors we did not find it meaningful to prepare a protocol in advance as we were only interested in the change in the number of complicated and uncomplicated appendicitis during the two study periods, irrespective of how they were defined, assuming that the criteria used did not change between these periods. We excluded all studies with indications of a change in management during the pandemic period.

### Statistical analyses

**Fig. 1:**
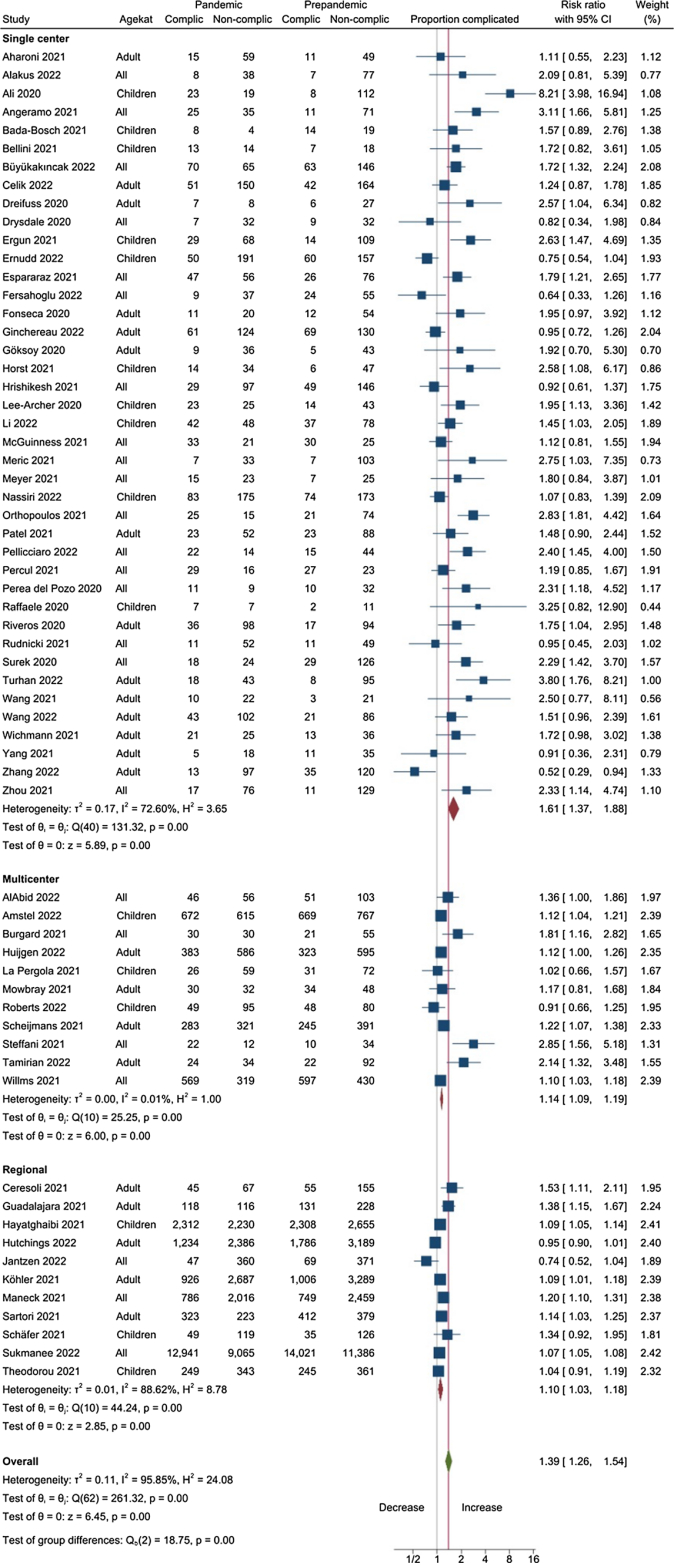
Forest plot of the meta-analysis of the change in proportion of complicated appendicitis during the Covid-pandemic compared with the pre-pandemic period, expressed as risk ratio

The extracted data were analyzed in three ways:

1. We did a random effects meta-analysis of the differences in the proportion of complicated appendicitis between the pandemic and pre-pandemic periods—expressed as the risk ratio (RR), visualized in a forest plot (Fig. 1).
2. We did separate random effects meta-analyses of the ratio of the number of patients with complicated and uncomplicated appendicitis between the pandemic and pre-pandemic periods, expressed as the incidence ratio (IR) with exact confidence limits, visualized in forest plots. The number of patients observed during the pandemic period is compared with the numbers in previous year(s). Separate analyses were performed for subsets according to the type of origin of the study (single- or multi-center or regional) and the included population (children, adult, or all ages).
3. We estimated the incidence ratio (IR) of complicated and uncomplicated appendicitis associated with the Covid-19 pandemic using a multilevel negative binomial regression model. The model was estimated using Bayesian inference and weighted by the square root of each reports sample size. Included in the model were covariates for origin of study (single- or multi-center or regional) and the included population (pediatric, adult, or all ages). The marginal posterior medians are reported as point estimates, alongside a compatibility interval (CoI) represented by the 2.5% and 97.5% percentiles of the posterior distribution.

Heterogeneity was analyzed with the I^2 test. Presence of bias was analyzed with funnel-plots and the egger test. Statistical analyses were performed using Stata 17 (StataCorp, College Station, TX, USA), R 4.0.4, and CmdStan 2.30.1.

## Results

### Descriptive results of selected studies

The primary search resulted in 836 unique references. After an initial screening of title and abstracts there remained 245 reports. A review of the full text resulted in exclusion of another 182 reports, leaving 63 reports with 100,059 patients from 25 countries (22,162 patients with complicated and 23,853 with uncomplicated appendicitis during the pandemic period, compared with 23,737 patients with complicated and 30,307 with uncomplicated appendicitis during the pre-pandemic period (Tables 1 and 2). The majority (41) were reports from single centers, 11 from multi-centers and 11 from geographic regions. Some 24 reports include all ages, 23 include only adults and 16 only pediatric patients.

**Table 1:** Characteristics of the included studies

Main author	Study reference	Age	Source	Country	Pandemic period	No. days	Pre-pandemic period
Aharoni (2021)	[51]	Adult	Single center	Israel	2020-03-01–2020-04-30	61	Same 2019
Al Abid (2022)	[52]	All	Multicenter	Australia/New Zealand	2020-03-15–2020-05-15	61	Same 2019
Alakus (2022)	[53]	All	Single center	Turkey	2020-03-11–2020-05-11	61	Same 2019
Ali (2020)	[54]	Children	Single center	Pakistan	2020-03-01–2020-05-30	92	Same 2019
Amstel (2022)	[28]	Children	Multicenter	International	3 months from start of covid	91	Same 2019
Angeramo (2021)	[55]	All	Single center	Argentina	2020-04-01–2020-08-31	152	Same 2018, 2019
Bada-Bosch (2021)	[56]	Children	Single canter	Spain	2020-03-13–2020-05-09	57	Same 2019
Bellini (2021)	[57]	Children	Single center	Spain	2020-03-01–2020-05-03	63	Same 2017, 2018, 2019
Burgard (2021)	[58]	All	Multicenter	Switzerland	2020-03-12–2020-06-06	86	Same 2017, 2018, 2019
Büyükakıncak (2022)	[59]	All	Single center	Turkey	2020-03-15–2020-05-15	61	Same 2019
Celik (2022)	[60]	Adult	Single center	Turkey	2020-03-11–2020-09-11	184	Same 2019
Ceresoli (2021)	[61]	Adult	Multicenter	Italy	2020-03-01–2020-04 30	60	Same 2018, 2019
Dreifuss (2020)	[62]	Adult	Single center	Argentina	2020-04-01–2020-04-30	29	Same 2018, 2019
Drysdale (2020)	[63]	All	Single center	Australia	2020-03-30–2020-05-17	49	Same 2019
Ergun (2021)	[64]	Children	Single center	Turkey	2020-03-11–2020-09-30	203	Same 2012, 2018, 2019
Ernudd (2022)	[65]	Children	single center	Sweden	2020-03-16–2020-06-16	92	Same 2017, 2018, 2019
Espararaz (2021)	[66]	All	Single center	USA	2020-03-01–2020-05-31	91	Same 2019
Fersahoglu (2022)	[29]	All	Single center	Turkey	2020-03-11–2020-06-01	50	Same 2019
Fonseca (2020)	[67]	Adult	Single center	Brazil	2020-03-01–2020-04-30	61	Same 2019
Ginchereau (2022)	[68]	Adult	Single center	Canada	2020-03-13–2020-06-30	109	Same 2019
Guadalajara (2021)	[69]	Adult	Multicenter	Spain	2020-03-14–2020-05-02	49	Same 2019
Göksoy (2020)	[70]	Adult	Single center	Turkey	2020-03-15–2020-05-15	61	Same 2019
Hayatghaibi (2021)	[34]	Children	Multicenter	USA	2020-03-01–2020-05-31	91	Same 2017, 2018, 2019
Horst (2021)	[31]	Children	Single center	USA	2020-03-01–2020-05-31	91	Same2019
Hrishikesh (2021)	[71]	All	Single center	UK	2020-03-01–2020-06-05	97	Same 2019
Huijgen (2022)	[72]	Adult	Multicenter	Netherlands	2020-03-12–2020-05-31: 2020-10-14–2020-12-31	151	Same 2019
Hutchings (2022)	[73]	Adult	Regional	England	2020-03-11–2020-05-12	63	Same2019
Jantzen (2022)	[74]	All	Regional	Denmark	2020-03-23–2020-04-19	27	Same 2017, 2018, 2019
Köhler (2021)	[4, 43]*	Adult	Regional	Germany	2020-03-01–2020-06-30	121	Same 2017, 2018, 2019
La Pergola (2021)	[75]	Children	Multicenter	Italy	2020-02-20–2020-04-20	60	Same 2017, 2018, 2019
Lee-Archer (2020)	[76]	Children	Single center	Australia	2020-03-16–2020-05-05	50	Same 2019
Li (2022)	[77]	Children	Single center	USA	2020-03-01–2020-06-30	121	Same 2019
Maneck (2021)	[26]	All	Regional	Germany	2020-03-16–2020-04-26	41	Same 2018,2019
McGuinness (2021)	[78]	All	Single center	New Zealand	2020-02-28–2020-06-08	101	Same 2019
Meric (2021)	[33]	All	Single center	Turkey	2020-03-13–2020-05-20	70	Same 2019
Meyer (2021)	[79]	All	Single center	Germany	2020-03-01–2020-08-31	183	Same 2018, 2019
Mowbray (2021)	[32]	Adult	Multicenter	Wales	2020-04-01–2020-04-30	29	Same 2018, 2019
Nassiri (2022)	[80]	Children	Single center	USA	2020-03-23–2020-08-31	161	Same 2019
Orthopoulos (2021)	[81]	All	Single center	USA	2020-03-16–2020-04-30	45	Same 2018, 2019
Patel (2021)	[35]	Adult	Single center	USA	2020-03-15–2020-05-32	77	Same 2019
Pellicciaro (2022)	[82]	All	Single center	Italy	2020-03-10–2021-03-10	365	Same 2019, 2020
Percul (2021)	[83]	All	Single center	Argentina	2020-03-20–2020-08-20	153	Same 2019
Perea del Pozo (2020)	[84]	All	Single center	Spain	2020-03-11–2020-04-17	37	Same 2019
Raffaele (2020)	[85]	Children	Single center	Italy	2020-02-01–2020-05-30	111	Same 2019
Riveros (2020)	[86]	Adult	Single center	Chile	2020-03-16–2020-08-16	153	Same 2019
Roberts (2022)	[87]	Children	Multicenter	Australia/New Zealand	2020-03-01–2020-04-30	60	Same 2018, 2019
Rudnicki (2021)	[88]	All	Single center	Israel	2020-03-01–2020-04-30	60	Same 2019
Sartori (2021)[55]	[89]	Adult	Regional	Italy	2020-03-01–2020-04-30	60	Same 2019
Scheijmans (2021)	[38]	Adult	multicenter	Netherlands	2020-03-15–2020-04-30	46	Same 2019
Schäfer (2021)	[44]	Children	Multicenter	Germany	2020-03-20–2020-05-31	72	Same 2018, 2019
Steffani (2021)	[45]	All	Multicenter	Germany	2020-03-15–2020-05-15	61	Same 2018, 2019
Sukmanee (2022)	[90]	All	Regional	Thailand	2020-03-01–2020-06-31	121	Same 2019 2021
Surek (2020)	[91]	All	Single center	Turkey	2020-03-14–2020-05-15	62	Same 2019
Tamirian (2022)	[37]	Adult	Multicenter	USA	2020-03-22–2020-05-31	80	Same 2019
Theodorou (2021)	[92]	Children	Multicenter	USA	2020-03-19–2020-09-19	184	Same 2019
Turhan (2022)	[93]	Adult	Single center	Turkey	2020-03-11–2020-05-21	71	Same 2019
Wang (2021)	[94]	Adult	Single center	Japan	2020-01-01–2020-05-30	153	Same 2018, 2019
Wang (2022)	[95]	Adult	Single center	Taiwan	2020-01-01–2020-06-30	182	Same 2017, 2018, 2019
Wichmann (2021)	[46]	Adult	Single center	Germany	2020-03-16–2020-05-31	76	Same 2018, 2019
Willms (2021)	[27]	All	Multicenter	Germany	2020-02-25–2020-05-05	70	Same 2019
Yang (2021)	[96]	Adult	Single center	China	2020-01-01–2020-09-30	151	Same 2019
Zhang (2022)	[97]	Adult	Single center	China	2020-02-01–2020-06-30	150	Same 2019
Zhou (2021)	[98]	All	Single center	China	2020-01-27–2020-03-31	64	Same 2019

*ref [43] gives an overall description of study. Number of patients are extracted from ref [4]

**Table 2:** Distribution of number of patients with complicated and uncomplicated appendicitis, and information about significant prehospital delay in the included reports

Main author	Study reference	Pandemic period			Pre-pandemic period			Delay*
		No. complicated	No. uncomplicated	Proportion complicated	No. complicated	No. uncomplicated	Proportion complicated	
Aharoni (2021)	[51]	15	59	20.3	11	49	18.3	0
AlAbid (2022)	[52]	46	56	45.1	51	103	33.1	0
Alakus (2022)	[53]	8	38	17.4	7	77	8.3	1
Ali (2020)	[54]	23	19	54.8	8	112	6.7	–
Amstel (2022)	[28]	672	615	52.2	669	767	46.6	–
Angeramo (2021)	[55]	25	35	42.7	11	71	13.4	1
Bada-Bosch (2021)	[56]	8	4	66.7	14	19	42.4	–
Bellini (2021)	[57]	13	14	48.1	7	18	28.0	–
Burgard (2021)	[58]	30	30	50.0	21	55	27.6	1
Büyükakıncak (2022)	[59]	70	65	51.9	63	146	30.1	–
Celik (2022)	[60]	51	150	25.4	42	164	20.4	–
Ceresoli (2021)	[61]	45	67	40.2	55	155	26.2	–
Dreifuss (2020)	[62]	7	8	46.7	6	27	18.2	1
Drysdale (2020)	[63]	7	32	17.9	9	32	22.0	–
Ergun (2021)	[64]	29	68	29.9	14	109	11.4	1
Ernudd (2022)	[65]	50	191	20.7	60	157	27.6	–
Espararaz (2021)	[66]	47	56	45.6	26	76	25.5	–
Fersahoglu (2022)	[29]	9	37	19.6	24	55	30.4	–
Fonseca (2020)	[67]	11	20	35.5	12	54	18.2	1
Ginchereau (2022)	[68]	61	124	33.0	69	130	34.7	0
Guadalajara (2021)	[69]	118	116	50.4	131	228	36.5	
Göksoy (2020)	[70]	9	36	20.0	5	43	10.4	–
Hayatghaibi (2021)	[34]	2312	2230	50.9	2308	2655	46.5	–
Horst (2021)	[31]	14	34	29.2	6	47	11.3	1
Hrishikesh (2021)	[71]	29	97	23.0	49	146	25.1	–
Huijgen (2022)	[72]	383	586	39.5	323	595	35.2	0
Hutchings (2022)	[73]	1234	2386	34.1	1786	3189	35.9	–
Jantzen (2022)	[74]	47	360	11.5	69	371	15.7	–
Köhler (2021)	[4, 43]	926	2687	25.6	1006	3289	23.4	–
La Pergola (2021)	[75]	26	59	30.6	31	72	30.1	0
Lee-Archer (2020)	[76]	23	25	47.9	14	43	24.6	–
Li (2022)	[77]	42	48	46.7	37	78	32.2	1
Maneck (2021)	[26]	786	2016	28.1	749	2459	23.3	–
McGuinness (2021)	[78]	33	21	61.1	30	25	54.5	–
Meric (2021)	[33]	7	33	17.5	7	103	6.4	–
Meyer (2021)	[79]	15	23	39.5	7	25	21.9	0
Mowbray (2021)	[32]	30	32	48.4	34	48	41.5	1
Nassiri (2022)	[80]	83	175	32.2	74	173	30.0	0
Orthopoulos (2021)	[81]	25	15	62.5	21	74	22.1	0
Patel (2021)	[35]	23	52	30.7	23	88	20.7	0
Pellicciaro (2022)	[82]	22	14	61.1	15	44	25.4	1
Percul (2021)	[83]	29	16	64.4	27	23	54.0	0
Perea del Pozo 2020	[84]	11	9	55.0	10	32	23.8	–
Raffaele 2020	[85]	7	7	50.0	2	11	15.4	–
Riveros 2020	[86]	36	98	26.9	17	94	15.3	–
Roberts (2022)	[87]	49	95	34.0	48	80	37.5	0
Rudnicki (2021)	[88]	11	52	17.5	11	49	18.3	1
Sartori (2021) w	[89]	323	223	59.2	412	379	52.1	–
Scheijmans (2021)	[38]	283	321	46.9	245	391	38.5	1
Schäfer (2021)	[44]	49	119	29.2	35	126	21.7	–
Steffani (2021)	[45]	22	12	64.7	10	34	22.7	0
Sukmanee (2022)	[90]	12,941	9065	58.8	14,021	11,386	55.2	–
Surek 2020	[91]	18	24	42.9	29	126	18.7	–
Tamirian (2022)	[37]	24	34	41.4	22	92	19.3	1
Theodorou (2021)	[92]	249	343	42.1	245	361	40.4	0
Turhan (2022)	[93]	18	43	29.5	8	95	7.8	1
Wang (2021)	[94]	10	22	31.3	3	21	12.5	0
Wang (2022)	[95]	43	102	29.7	21	86	19.6	1
Wichmann (2021)	[46]	21	25	45.7	13	36	26.5	1
Willms (2021)	[27]	569	319	64.1	597	430	58.1	0
Yang (2021)	[96]	5	18	21.7	11	35	23.9	1
Zhang (2022)	[97]	13	97	11.8	35	120	22.6	0
Zhou (2021)	[98]	17	76	18.3	11	129	7.9	–

*Delay indicates that duration of symptoms on arrival was significantly different between the study periods (= 1), or not (= 0). A dot (.) indicates information on delay was not presented

The median duration of the pandemic study period was 72 days (range 27–365). The reference period was the same dates in previous year(s) as the for the pandemic period, in 42 reports from 2019, 12 reports included 2018 and 2019, and 9 reports included three years (2017–2019). For the reports with more than one year for the reference period we used the mean number of patients over the years for the analyses. The prehospital duration of symptoms was significantly longer, suggesting delay in 17 of 33 studies that reported this information (Table 2).

### Impact on proportion of complicated appendicitis

The Covid pandemic was associated with an increased risk ratio (RR) of the proportion of complicated appendicitis compared to the pre-pandemic period (RR 1.39, confidence interval (CI) 1.25, 1.53) (Fig. 1 and Table 3). This was most marked for the single center reports (RR 1.61, CI 1.37, 1.88), while the increase was smaller in the multi-center and regional based reports (RR 1.13, CI 1.08, 1.19). The meta-analysis shows strong heterogeneity within and between the groups reflecting the differences in definitions of outcome, in level of restrained access and size of reports. This is also evident from the funnel plot, Supplementary Fig. 2. The Egger test is strongly significant (*p* < 0.001) suggesting small study bias.

**Table 3:** Impact of the Covid-pandemic on the proportion of complicated appendicitis reported as the risk ratio (RR), and on the incidence of complicated and uncomplicated appendicitis, comparing the pandemic with the pre-pandemic period, reported as incidence ratio (IR) with 95% confidence interval (CI)

Study base	Number of reports	Proportion complicated		Complicated appendicitis		Uncomplicated appendicitis	
		Risk ratio	95% CI	Incidence ratio	95% CI	Incidence ratio	95% CI
All types of study base	63	1.39	1.25, 1.53	1.15	1.04, 1.27	0.66	0.59, 0.73
Single-center	41	1.61	1.37, 1.88	1.27	1.09, 1.49	0.62	0.53, 0.72
Multi-center	11	1.14	1.09, 1.19	1.08	0.97, 1.19	0.70	0.57, 0.86
Regional	11	1.11	1.03, 1.18	0.91	0.82, 1.01	0.75	0.65, 0.87
Multicenter + Regional	22	1.13	1.08, 1.19	0.98	0.90, 1.07	0.73	0.65, 0.82
*Agecategory*							
Children	16	1.44	1.11, 1.85	1.29	1.05, 1.57	0.73	0.59, 0.92
Adult	24	1.49	1.25, 1.78	1.10	0.94, 1.29	0.57	0.48, 0.69
All ages	23	1.27	1.13, 1.42	1.11	0.93, 1.33	0.71	0.61, 0.82
*Delay*							
No delay	16	1.21	1.02, 1.44	1.11	0.93, 1.33	0.79	0.65, 0.95
Delay present	17	1.75	1.46, 2.11	1.40	1.17, 1.66	0.59	0.49, 0.70

Results are presented for all reports overall and for subgroups. All studies show large degree of heterogeneity with an I^2 over 90% for all analyses

### Impact on number of complicated and uncomplicated appendicitis

**Fig. 2:**
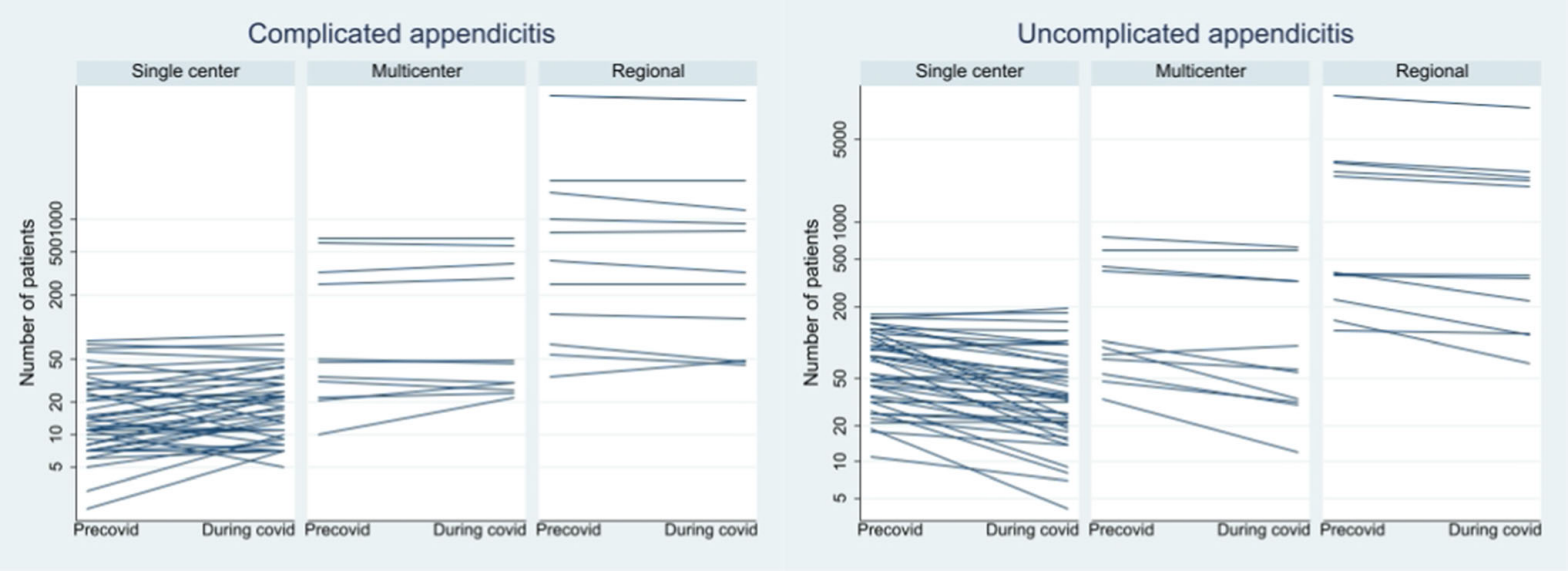
Graphical presentation linking the observed number of patients during the pre-pandemic and pandemic periods for each report, according to type of study base and severity of appendicitis. The *y*-axis is logarithmic. The smaller studies show large heterogeneity and also pattern suggesting regression to the mean. The larger studies show mainly stable incidence of complicated appendicitis and a trend toward decreasing incidence of uncomplicated appendicitis

The change in the absolute number of patients with complicated and uncomplicated appendicitis between the pre-pandemic and pandemic periods according to the type of report is graphically shown in Fig. 2. These graphs show a clear difference in trends between type of source. For complicated appendicitis the smaller, single center studies, show a pattern that suggest regression to the mean with some decreasing and some increasing numbers. This can be expected given the small numbers and short observation period. For most of the studies the numbers of complicated appendicitis remain at a stable level. For the numbers of uncomplicated appendicitis there is a marked trend toward a lower number during the pandemic period compared with the pre-pandemic period in most studies.

**Fig. 3:**
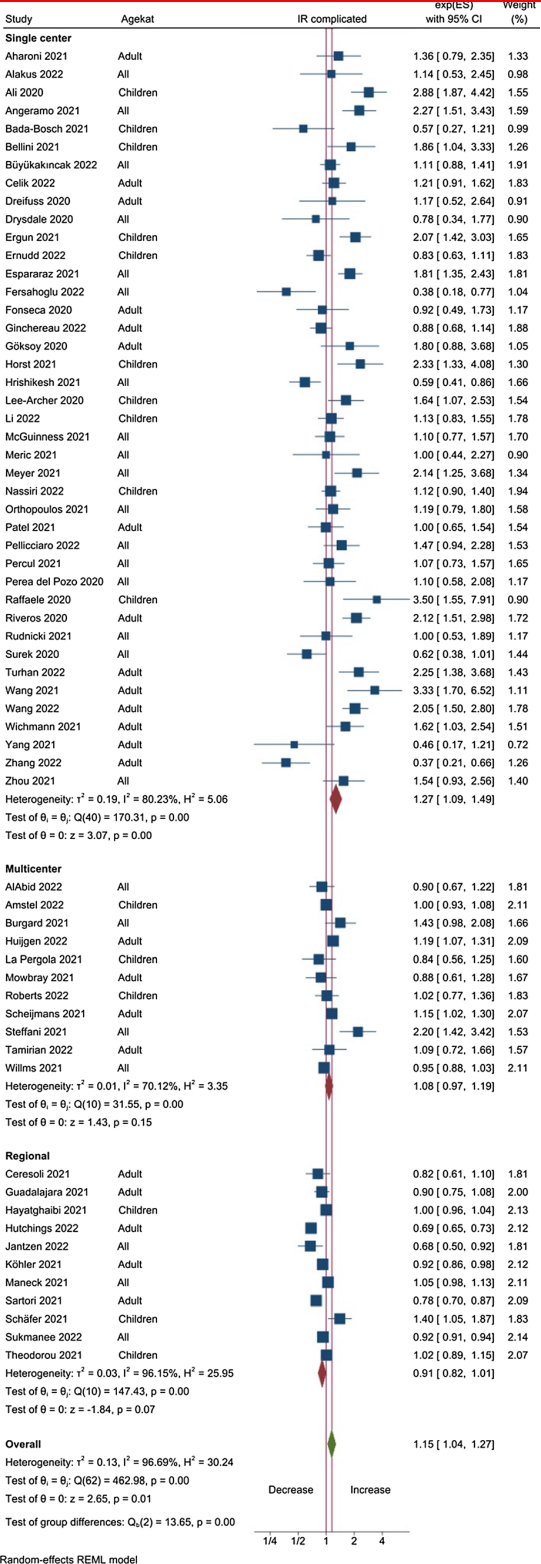
Forest plot of the meta-analysis comparing the number of complicated appendicitis before and during the Covid-19 pandemic, expressed as the incidence ratio. For the larger studies the IR is close to one, supporting no impact on the incidence of complicated appendicitis during the pandemic

The meta-analysis comparing the number of complicated appendicitis before and during the Covid-19 pandemic period, expressed as the Incidence Ratio (IR), shows an increase overall (IR 1.15, CI 1.04, 1.27), most marked in children (IR 1.29, CI 1.05, 1.57) but with a marked difference between the single center and the other source types. When the reports from multi-center and regional are combined there is no increase in the number of complicated appendicitis (IR 0.98, CI 0.90, 1.07) (Table 3, Fig. 3).

**Fig. 4:**
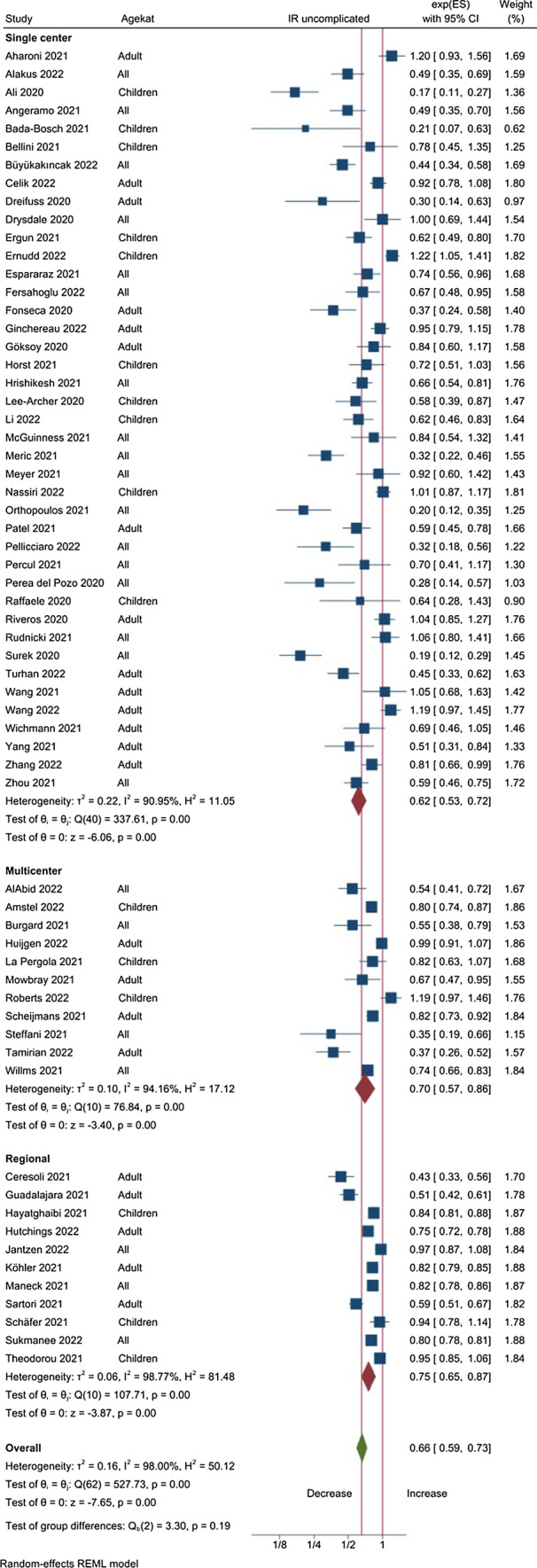
Forest plot of the meta-analyses comparing the number of uncomplicated appendicitis before and during the Covid-19 pandemic, expressed as the incidence ratio. For the larger studies the incidence ratio is significant lower than 1.0, supporting a decrease in the incidence of uncomplicated appendicitis during the pandemic

For uncomplicated appendicitis the meta-analysis shows a marked decrease in all the subsets with an IR of 0.66 (CI 0.59; 0.73) overall, with the strongest decrease in the single center studies (IR 0.62, CI 0.53: 0.73) and in reports based on adults (IR 0.57, CI 0.48; 0.69) (Table 3, Fig. 4).

Finally, the multilevel negative binomial regression model revealed that the posterior probability that the IR for complicated appendicitis associated with the Covid-19 pandemic was greater than 1 was 98.5% (median adjusted IR = 1.13, Compatibility Interval (CoI) = 1.01, 1.26), after having adjusted for both origin of study and included population. This posterior probability is the product of our Bayesian analysis [24], and can be interpreted as the proportion of all possible IR estimates which are, given the data and the model, compatible with the assertion that the association is greater than the null (IR = 1)—i.e., the probability that there was a difference between the two points in time. Similarly, the posterior probability that the IR for uncomplicated appendicitis associated with the Covid-19 pandemic was *less* than 1 was > 99.9% (median adjusted IR = 0.64, CoI = 0.57, 0.72). When removing single center studies, the posterior probability that the IR for complicated appendicitis associated with the Covid-19 pandemic was greater than 1 was 59.0% (median adjusted IR = 0.99, 95% CoI = 0.98, 1.10), i.e., when excluding single center studies the probability that there was a difference between the two points in time was much lower. For uncomplicated appendicitis on the other hand, the posterior probability that the IR associated with Covid-19 pandemic was less than 1 remained high, > 99.9%, with a median adjusted IR of 0.71 (95% CoI = 0.61, 0.82).

Overall, our analyses suggest strong evidence that uncomplicated appendicitis decreased substantially during the pandemic whereas the pandemic had little impact on the incidence of complicated appendicitis.

### Association with delay

Delay was associated with higher proportion of complicated appendicitis, and higher incidence ratio of complicated appendicitis but had also the lowest incidence ratio of uncomplicated appendicitis (Table 3).

### Quality of the reports and risk for bias

The studies show important heterogeneity with an I^2 statistic > 90% in all analyses. This is also evident from funnelplots of the IR of complicated and uncomplicated appendicitis (Supplementary Fig. 3 and 4). Possible reasons for this may be differences in level of limitation in access to health care during the pandemic (the exposure) and in definitions for grading the severity of appendicitis (the outcome). The sample size and length of the inclusion period also varies. Because of this heterogeneity a formal estimation of the overall effect of the pandemic on the incidence and outcome of appendicitis is not possible. However, the reports internal validity should be acceptable as it seems likely that the definition of the severity of appendicitis should be identical in the pandemic and reference periods for each of the type of sources, and the catchment population should also be identical as we have excluded studies showing indications suggesting changes in referrals. This is especially true for the regional studies, which should have higher attention for the interpretation of the results.

## Discussion

In accordance with many previous reports, we found an increase in the proportion of complicated appendicitis during the first wave of the Covid-19 pandemic, compared with a representative pre-pandemic period. A common interpretation is that this indicates progression of severity because of delay. However, an increased proportion of complicated appendicitis (i.e., “perforation rate”) can be the result of both an increase in the number of complicated appendicitis as well as a decrease in the number of uncomplicated appendicitis [25].

In this meta-analysis, we found that the Covid-19 pandemic was associated with a consistent decrease in the number of uncomplicated appendicitis and a stable incidence of complicated appendicitis in the more reliable study sources. The increased proportion of complicated appendicitis during the pandemic is thus not a sign of an increased risk for the progression of the severity due to the limited access to health care, but the results in this meta-analysis rather suggest an increase in spontaneous resolution of uncomplicated appendicitis. This interpretation is also proposed in many reports [7, 26–30, 30–38].

Early diagnosis and treatment is by tradition regarded as important for preventing the progression of the inflammation that will eventually lead to perforation if left untreated. Due to limited data of questionable quality we could not analyze the direct impact of prehospital duration of symptoms, but the overall impact of the restrained access to health care in the present study support previous reports showing that an association between prehospital delay and the proportion of complicated appendicitis may be explained by selection of complicated cases with time as the uncomplicated cases resolve undiagnosed [9, 39, 40]. This is also supported by the safety of expectant management with in-hospital observation in up to 36 h or deferring operations to daytime [41, 42].

When interpreting the findings of this meta-analysis several limitations should be borne in mind. The studies show important heterogeneity. This is expected as there are probably large differences in the exposure (decrease in access to health care) as well as the outcome (definition of severity of appendicitis) between the studies. However, we can assume that within each report the definitions should be consistent over the two study periods, and consequently the direction of the changes should be consistent, except for smaller studies where regression toward the mean is a possible explanation. The results of the larger multi-center and regional studies also show a more homogenous direction of the association with a decrease in the incidence of uncomplicated and stable incidence of complicated appendicitis during the pandemic.

All included reports are retrospective observational studies from many different types of sources. To avoid bias due to seasonality we have only included reports that use the same calendar period for comparison between the pandemic and reference periods. We have excluded some reports with large differences in age and sex distribution between the study periods which suggest changes in the catchment population due to changed referral pathways. We have also excluded reports with large changes in the use of non-operative treatment with antibiotics for uncomplicated appendicitis. We have included six reports from Germany [26, 27, 43–46]. Some of these studies may be partly overlapping. Due to limited information on the geographical location of the included clinics, we cannot adjust for this possible overlap.

The findings in this study support the growing body of evidence showing that complicated and uncomplicated appendicitis are two different entities, one that progress to complicated appendicitis early and another that resolve spontaneously [8, 40, 47]. This has important principal implications for the management of patients with suspicion of appendicitis also outside the pandemic. The primary goal is the early detection and treatment of complicated appendicitis, whereas the detection and treatment of uncomplicated appendicitis are less urgent as they rarely progress to perforation but may heal spontaneously, as suggested by the results in this meta-analysis. The increased use of diagnostic imaging during the last decades has led to the increased detection of mild, uncomplicated appendicitis that was previously allowed to heal undetected [19, 31–33]. This increased detection and treatment of mild, uncomplicated appendicitis has been associated with a decrease in the proportion of complicated appendicitis but has not had any impact on the incidence rate of complicated appendicitis [48]. Spontaneous resolution of uncomplicated appendicitis has also an implication on the current trend toward antibiotics treatment of uncomplicated appendicitis. Two placebo-controlled studies show same healing rate irrespective if the patients were given placebo or antibiotics [49, 50]. This strongly suggests that the effect seen in studies of antibiotics treatment of uncomplicated appendicitis is to a large extent the result of spontaneous healing. This supports expectant management of patients with uncomplicated appendicitis.

## Conclusion

We confirm previous reports of an increase in the proportion of complicated appendicitis during the Covid-19 pandemic. However, a more detailed analysis shows that this is the result of a substantial decrease in the incidence of uncomplicated appendicitis, whereas the incidence of complicated appendicitis remained stable.

This result suggests that the restrained access to health care during the Covid-19 pandemic resulted in an increase in resolution of undetected uncomplicated appendicitis. This support previous indications that complicated and uncomplicated appendicitis are different entities, and that uncomplicated appendicitis may resolve without treatment. This has important principal implications on the management of patients with suspicion of appendicitis also outside the pandemic.

## Supplementary Information

Below is the link to the electronic supplementary material.

Supplementary file1 (DOCX 410 KB)
