# Supplementary material for: Spontaneous Resolution of Uncomplicated Appendicitis may Explain Increase in Proportion of Complicated Appendicitis During Covid-19 Pandemic: a Systematic Review and Meta-analysis
Source: World J Surg. 2023 May 4;47(8):1901–16. doi: 10.1007/s00268-023-07027-z (PMC10158710; doi:10.1007/s00268-023-07027-z)

Supplementary Figure 1. PRISMA flow diagram for the selection process of eligible studies


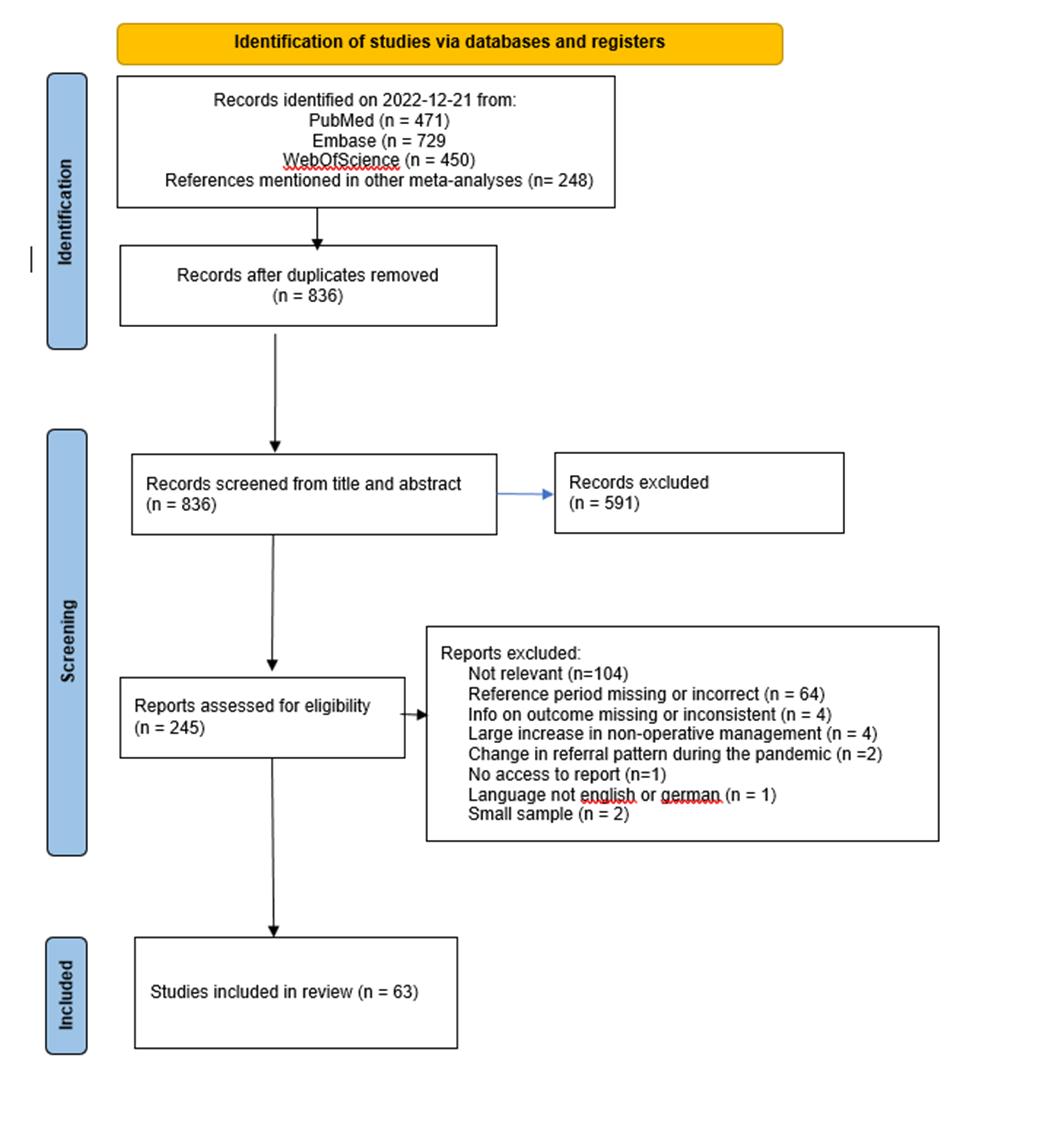


Supplementary Figure 2. Funnel plot of the Risk Ratio of the proportion of complicated appendicitis in 63 reports, according to study base (S=single center, M=multicenter, R=regional). It shows strong heterogeneity between studies and the Egger test is strongly significant suggesting small study bias.


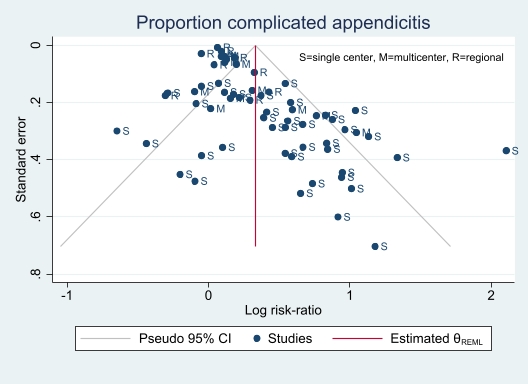


Supplementary Figure 3. Funnelplot of the impact of Covid-19 pandemic on the incidence of complicated appendicitis compared with the prepandemic period, expressed as the Incidence Ratio. The plot show large heterogeneity, especially in the smaller studies.


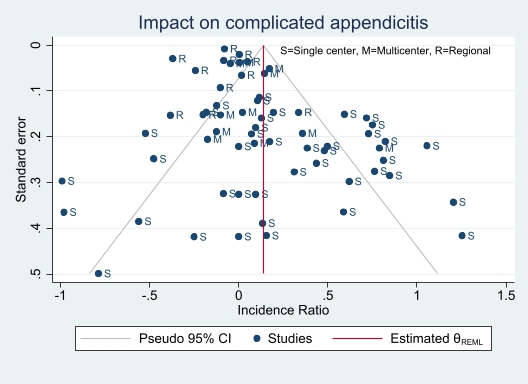


Supplementary Figure 4. Funnelplot of the impact of Covid-19 pandemic on the incidence of uncomplicated appendicitis compared with the prepandemic period, expressed as the Incidence Ratio. The plot shows large heterogeneity, especially in the smaller studies.


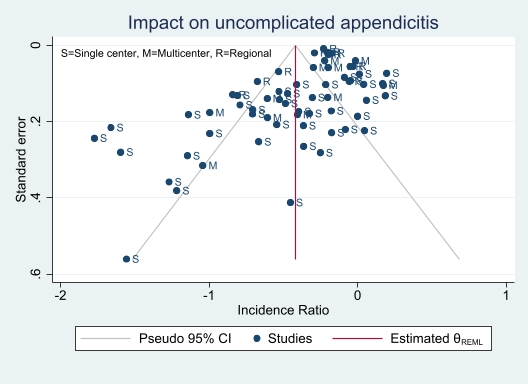

Supplement: Supplementary file 1 — Supplementary file1 (DOCX 410 KB) [file 268_2023_7027_MOESM1_ESM.docx]
